# Efficacy of belimumab monotherapy in high infectious risk patient affected by lupus nephritis

**DOI:** 10.1093/rap/rkab023

**Published:** 2021-04-17

**Authors:** Roberto D’Alessandro, Estrella Garcia Gonzalez, Bruno Frediani

**Affiliations:** Rheumatology Unit, Department of Medicine, Surgery and Neurosciences, University of Siena, Siena, Italy

**Key message**

- Belimumab could be an effective option for LN, in monotherapy.

Dear Editor, Lupus nephritis (LN) is the most common severe manifestation of Systemic Lupus Erythematosus (SLE). It develops in ∼40% of SLE patients, and treatment with mycophenolate mofetil (MMF) or cyclophosphamide (CYC) is indicated to induce remission [1]. Belimumab (BEL), a human monoclonal antibody inhibiting B cell activating factor (BAFF) is approved worldwide for the treatment of several SLE manifestations and when added to standard-of-care it may gradually reduce proteinuria and the risk for LN [1].

**Fig. 1:**
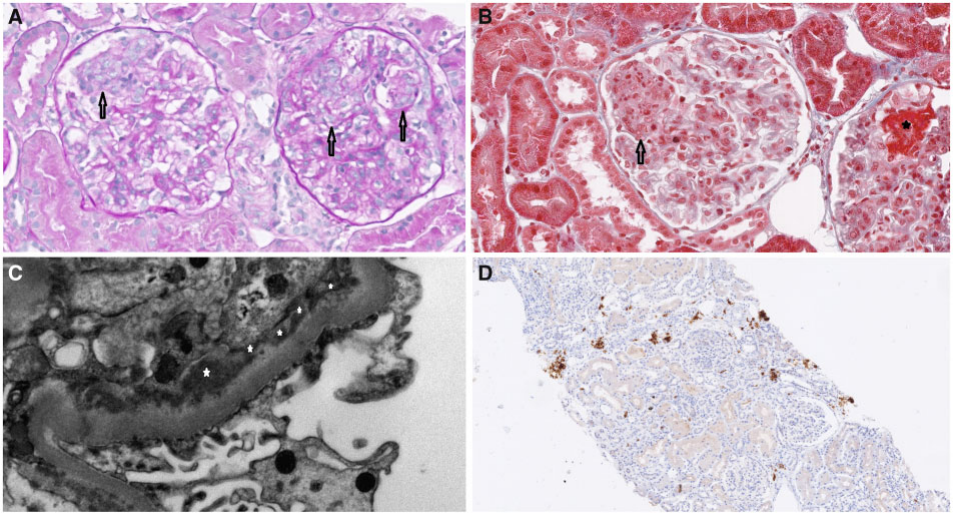
Renal biopsy specimens (**A**, **B**) The photomicrographs show segmental endocapillary proliferation (arrows) and necrosis (star) (**A**, periodic acid–Schiff, ×400; **B**, Masson’s Trichrome, ×400). (**C**) The electron micrograph shows massive subendothelial deposits (stars; ×2200). (**D**) Plasma cell-rich inflammatory infiltrate (immunohistochemistry, CD38^+^).

We herein report the case of a 35-year-old North African woman, who was diagnosed with SLE in 2011, with severe haematological and cutaneous involvement and initially treated with hydroxychloroquine (HCQ) 200 mg twice a day, MMF 1 g twice a day and prednisone 0.5 mg/kg/day. In 2015 she was hospitalized in the intensive care unit owing to severe Cytomegalovirus pneumonia pneumonia. MMF was discontinued, and after recovery, treatment with azathioprine (AZA) 100 mg/day and intravenous immunoglobulin 25 g/monthly was started on a background of HCQ. In March 2017 at follow-up, because of the evidence of proteinuria (2311 mg/24 h), hypocomplementaemia C3 (12 mg/dl) and a mild increase of serum creatinine (SCr) (1.20 mg/dl), she underwent a renal needle biopsy. Histology showed stage 4 Glomerulonephritis, characterized by segmental endocapillary proliferation, necrosis and subendothelial deposits (Fig. 1). Induction treatment with methylprednisolone pulses followed by i.v. CYC, 500 mg every 2 weeks for 3 months (Euro-Lupus Regimen), was performed with initial benefit. Maintenance treatment with AZA and HCQ was started. One month later, the patient presented signs of bone marrow toxicity, probably related to AZA, which was then interrupted. A further increase in proteinuria (2670 mg/24 h) and SCr (1.3 mg/dl) occurred, with a high titre of anti-dsDNA antibodies (ab) (>379 U/ml) and a SLEDAI of 20. In consideration of her high disease activity and after carefully considering her infectious risk on medical history, the patient was screened for BEL, started on January 2018 (10 mg/kg intravenous/monthly) on a background of HCQ 200 mg twice a day and prednisone 0.5 mg/kg/day. Six months later, proteinuria, SCr and anti-dsDNA ab decreased to 1330 mg/24 h, 0.92 mg/dl and 192 UI/ml, respectively, C3 was 18 mg/dl and SLEDAI 16, with prednisone tapered to 12.5 mg/day. In December 2018, after the last intravenous BEL infusion, the patient presented a persistent reduction of proteinuria, SCr and anti-dsDNA ab (1019 mg/24 h, 1.2 mg/dl and 129 U/ml, respectively) with a further improvement of C3 (24 mg/dl) and SLEDAI at 6. The patient was switched to subcutaneous BEL (200 mg/weekly), leading to a constant decrease and stabilization of the disease. In November 2019, a remission in renal function was achieved, which was maintained until the last follow-up in June 2020, reaching a proteinuria of 473 mg/24 h, SCr of 1 mg/dl, C3 of 14 mg/dl and a SLEDAI of 4. Prednisone was tapered to 5 mg/day. No side effects or infections were reported in the follow-up period of 30 months.

Our case shows how BEL determined a long-term renal remission of stage 4 LN, leading to a low disease activity on a background of HCQ and low dosage of corticosteroids, without any other immunosuppressant agent. It must be specified that the CYC regimen was completed 6 months before the beginning of BEL, and this might have contributed to increase the efficacy of BEL as maintenance therapy. Furthermore, although clinical and laboratory remission does not always correspond to histopathological remission in LN, a second renal biopsy was not performed in consideration of the risk-to-benefit ratio of the procedure and to avoid any complication.

BAFF seems to play a pivotal role in LN, as recently evidenced by Schwarting *et al*. [2], who demonstrated its expression both in cultured murine and human tubular epithelial cells and in renal biopsies in patients with LN, demonstrating a correlation with the histopathological activity index. Recently, the randomised, double-blind, placebo controlled phase 3 trial of intravenous belimumab in patients with active lupus nephritis (BLISS-LN) has showed how patients affected by LN who received BEL plus standard therapy (MMF and AZA) had a better renal response than those who received standard therapy alone, confirming some data previously reported [3, 4]. On the contrary, it must be pointed out that some evidence showed the onset of LN during BEL treatment in SLE patients who apparently did not present with renal involvement [5, 6]. BEL is not yet recommended as sole primary treatment for LN, and there are no data on whether BEL could play a role in patients who failed induction therapy or relapse [7, 8].

We here report a long-term efficacy of BEL in monotherapy as maintenance treatment after CYC induction in a LN patient. As for our patient, we suggest that BEL might be an effective option for the treatment of LN, particularly in those patients who present a high infective risk and a refractory response to immunosuppressant agents. Future trials are needed to confirm whether BEL could be used *in solo* in LN.

*Funding*: This research did not receive any specific grant from funding agencies in the public, commercial or not-for-profit sectors.

*Disclosure statement*: The authors have declared no conflicts of interest.
